# Epidemic Cerebro-Spinal Meningitis

**Published:** 1848-10

**Authors:** 


### ECLECTIC DEPARTMENT,

### AND SPIRIT OF THE MEDICAL PERIODICAL PRESS.

*Epidemic Cerebro-Spinal Meningitis.*—The following summary of facts which have been communicated in different journals, respecting the above singular epidemic, is presented by Dr. Condie, in an annual Report on the diseases of children, read at a stated meeting of the College of Physicians of Philadelphia. The Report is published among the *transactions of the College, from April to August,* 1848, *inclusive.* These transactions, a summary of which is issued semi-annually, are amongst the most valuable of the contributions to the medical periodical literature of this country, and it were much to be desired that other Medical Associations would pursue a similar course, first, in providing for stated reports on subjects embraced in the different departments of medical and surgical science, and, next, in their publication for the benefit of the profession at large. We shall avail ourselves, in behalf of our readers, of other extracts from the present "summary of transactions," as we have done heretofore with respect to other numbers.—Editor Buffalo Med. Journal.

"In the *New Orleans Med. and Surg. Journal* we find two communications, the one from Dr. B. F. White, of Whiteville, Tennessee, and the other from Dr. B. J. Hicks, of Vicksburgh, Mississippi, describing an epidemic which occurred in their respective vicinities during the past year, the identity of which with the epidemics of Cerebro-spinal meningitis that have prevailed in different portions of Europe, is rendered evident by the similarity of the leading symptoms, the class of patients who were chiefly its subjects, and the lesions of the brain and spinal marrow discovered in the examinations after death made by Dr. White.

In Tennessee, the disease made its appearance, towards the close of February, in the region of country extending along the Hatchie river from Bolivar to Estanaula. It was principally confined to children between the ages of six and fifteen years. The attack was ushered in by a sensation of chilliness, followed by moderate heat of the surface, and pain, commencing between the shoulders and extending to the occipital region, with rigidity of the posterior cervical muscles, retracting the head considerably backwards. Delirium supervened in an hour or two, with contraction of the pupils of the eye, or sometimes dilation of one pupil and contraction of the other; ptosis of the eyelids; ecchymosis under the eyes and on the surface of the body; rigidity of the abdominal muscles; spasmodic twitchings of the flexors of the extremities, and a disposition to a constant motion of the legs from side to side, alternately. There was difficulty in expanding the lungs, respiration chiefly through the nostrils, constipation, and sometimes retention of urine. Stertorous breathing coming on, death soon closes the scene. The disease runs its course in from fifteen to seventy-two hours. Dr. White has known one case in which the disease was protracted to the twelfth day.

Almost every plan of treatment was tried—bleeding, emetics, cathartics, cold douche, cupping, mercurials, blisters, followed by opium, quinine, and stimulants. Every case in which the attack commenced with symptoms of extreme violence terminated fatally. Three-fourths of the patients fell victims to the disease. Towards the last, however, as is the case in nearly all epidemics, the disease, to a certain extent, assumed a less malignant form.

Dr. White conceives that the change of the blood by the lungs becomes suspended, and that death is produced principally from *asphyxia.*

The disease commenced in Vicksburg about the 10th of January, 1846, and continued until the close of March.

The patients were attacked with groaning, muttering delirium, chilly sensations, pallid countenance, cold extremeties; these symptoms were soon followed by restlessness, flushed countenance, frequent pulse, wild and frantic expression of the eyes, and hot and dry skin. The patient screamed violently when touched or spoken to, was unable to answer questions correctly, to locate the pain or indicate the suffering organs.—Whilst sleeping, alternate pallor and flushings of the countenance were often observed, and violent inflammation of the right or left eye was not an unfrequent occurrence. On the second or third day the spinal muscles became very much contracted and rigid; in some instances to such an extent that the patients had considerable difficulty in swallowing even fluids; there was at the same time a loss of power in either the right or left upper and lower extremeties, followed by convulsions of great severity, instantly excited by touching or raising the inferior extremities. There was no evidence of lesion of the biliary or digestive organs; the urinary secretion was unnaturally copious and pellucid.

There was absolute regularity in the period at which the various symptoms appeared; in some cases the tetanic symptoms were observed as early as the first day of the attack, and in others not until as late as the tenth. In many of the violent cases, within six hours after the patients were attacked, *petechiæ* of large size made their appearance upon the arms, over the eyelids, and upon the inferior extremeties. These petechiæ disappeared on the fourth or fifth day, if the patients were not sooner cut off. In the most violent cases, death ensued on the fifth day of the attack; in other instances they survived from thirty to fifty days. But few cases recovered after the tetanic symptoms presented themselves. The mortality amounted to about 50 per. cent. on all attacked.

Blood-letting, stimulants, and anodynes were found equally unsuccessful. In vigorous constitutions, blood-letting, in the onset of the attack, followed by an active mercurial cathartic, was often of great service. In patients of a more delicate constitution, cupping along the whole length of the spine and early vesication were preferred. Revulsives to the extremities and iced cloths to the head, with the internal exhibition of the following, in table-spoonful doses every two hours, were found to be of advantage, ℞. Pulv. gum. camph. ʒj.; Potass. tart. antimon. grs. ij.; Mucil. gum. acaciæ, ℥vj.; M.—An enema composed of ʒij. Spir. terebinth., ʒij. tr. assafœtida, and half a pint of thin starch, was administered morning and evening, with a view of tranquilizing nervous distress and keeping the bowels gently open.

The following was found to be the most beneficial tonic, after the violence of the disease had abated, for relieving the inertia of the nervous system, which occurred in every instance of recovery. ℞. Iod. ferri. ℈j.; Iodin. grs. viij.; Iod. potassæ, ℥ij.; Syr. sarsapar. ʒiv.; M. Given in doses of a tea-spoonful every four hours, in a little water. It should be continued for some days, unless found to produce gastric distress; in which case some mild bitter vegetable infusion should be substituted.

We are presented with the post-mortem appearances in a single case examined by Dr. White. The body was not emaciated, a half an inch of fat covered the abdominal muscles. The posterior integuments of the head were swollen; both pupils dilated; on removing the calvarium, a considerable amount of blood flowed from the sinuses of the dura matter. The arachnoid membrane adhered with moderate firmness to the surface of the convolutions; whilst removing the brain from two to three ounces of clear transparent serum escaped from the ventricles. The brain appeared heavier than usual. The surface of the convolutions was much flattened. The base of the brain bore evident marks of inflammation. The membranes covering the medulla oblongata and the cerebellum, the right lobe more especially, were thickened and opaque, adhering, likewise, pretty firmly to the fissures of Sylvius. The membranes at the base were unusually vascular, but the substance of the brain itself was not much altered in color or in consistence. The membranes, more particularly around the third nerve of the right side, were thickened and more vascular than natural. On examining the superior surface of the brain, and separating the two hemispheres, they gave way inferiorly, from a softness of the lower part of the middle lobe of both hemispheres, and of a considerable portion of the corpus callosum. The corpora striata were slightly injected, and softened, particularly that of the right side. The lining membrane of the ventricles was not altered in color.

On dividing the vertebræ, a considerable quantity of fluid blood gushed out, the moment the interior of the canal was reached. It appeared to be perfectly flooded and engorged. The membranes were evidently thickened and highly vascular. The spinal marrow was not altered in appearance, but, if anything, somewhat softer than natural. The substance of the marrow itself was not injected.

The viscera of the thorax presented no appearance of disease.

The liver was perfectly engorged with blood—an incision being made through its structure, the blood could be squeezed from it as from a sponge. Its weight was 5½ pounds. The gall bladder was distended with a quantity of thick, black, bilious matter. The spleen was large, but contained little blood in comparison with the liver.

The kidneys were congested but otherwise healthy. The intestines contained a quantity of thin greenish matter. There were a few spots of ecchymoses in the lower two-fifths of the ileum. The small intestines contained a few large worms. The alimentary canal was otherwise healthy. The urinary bladder bore no mark of disease.

Dr. Hicks gives also an account of the post-mortem examination in one case, but it is so deficient in detail that nothing satisfactory can be derived from it.

The same form of disease prevailed also, during the last year, at Rocheport, Boon county, Missouri. In a letter addressed to Professor Dunglison, (*Medical Examiner, Vol.* 10, *p.* 604.) Dr. W. C. Phillips, thus describes the disease as it appeared in the last mentioned locality.

It occurred in persons of all ages, but most commonly in children from 10 to 15 years. The first indication of an attack was often a pain in the hand, foot, arms, legs, eye, brain, lungs, stomach or bowels. Sometimes one, or at others a number of these locations at the same time, were primarily affected. In some cases there was a sensation as of cords drawing at the back of the neck, producing stiffness. In one case, there was a contraction of the occipito frontalis, and of the muscles of the face. In many instances there was so much soreness of the surface as to render the smallest amount of clothing intolerable. The disease was usually ushered in with a chill, of variable intensity, succeeded by a re-action, differing in intensity, and this re-action was followed, in from 3 to 12 hours, by a second chill. In many cases there was excruciating pain in the arms, legs, and other parts of the body, and this pain, when located in the limbs, was often accompanied with swelling of and stiffness and inability to move the joints and more or less loss of the use of the limbs. The pains were sometimes permanent, but at others, shifting from one point to the other. The neck was often drawn backwards or forwards to an angle of about 45° from its natural position, and so rigidly fixed, that it would break short off before it could be forced to resume its proper position—it was at the same time, considerably swollen. The external surface was sometimes beautifully spotted; in a few cases there was an exanthematous eruption. The disease was attended by either profound coma, wild and furious delirium, su sultus tendinum, apoplexy or paralysis. In one case there was blindness of one eye, accompanied with permanent pain in the head, and swelling of the joints. The lungs may or may not be implicated. The stomach was often affected with nausea and vomiting. The bowels were not particularly affected. Trismus occurred in one case. In other cases there was an inability to swallow anything, from the swollen and painful condition of the larynx pharynx.

Five-sixths of the cases terminated fatally.—In many instances the fatal event occurs almost at the very onset of the disease; in general, death takes place in from six to twenty-four hours.

Dr. Phillips gives no account of the lesions detected upon a post-mortem examination.

